# Biobanking of Exosomes in the Era of Precision Medicine: Are We There Yet?

**DOI:** 10.3390/ijms17010013

**Published:** 2015-12-24

**Authors:** Edna M. Mora, Silvia Álvarez-Cubela, Elisa Oltra

**Affiliations:** 1 Department of Surgery, School of Medicine, Medical Sciences Campus, University of Puerto Rico, San Juan 00936, Puerto Rico; edna.mora2@upr.edu; 2 University of Puerto Rico Comprehensive Cancer Center, San Juan 00936, Puerto Rico; 3 Diabetes Research Institute, University of Miami, Miami, FL 33136, USA; salvarez@med.miami.edu; 4 Facultad de Medicina, Universidad Católica de Valencia “San Vicente Mártir”, Valencia 46001, Spain; 5 Instituto Valenciano de Patología (IVP) de la Universidad Católica de Valencia “San Vicente Mártir”, Centro de Investigación Príncipe Felipe (CIPF), Valencia 46012, Spain

**Keywords:** exosome, exogram, biomarker, biobank, precision medicine

## Abstract

The emerge of personalized medicine demands high-quality human biospecimens with appropriate clinical annotation, especially in complex diseases such as cancer, neurodegenerative, cardiovascular, and metabolic alterations in which specimen heterogeneity and individual responses often complicate the development of precision therapeutic programs. In the growing field of extracellular vesicles (EVs) research, exosomes (EXOs)—a particular type of EVs—have been proposed as an advantageous diagnostic tool, as effective delivery vehicles and as therapeutic targets. However, the lack of consensus on isolation methods and rigorous criteria to characterize them puts the term EXO into question at the time that might explain some of the controversial results found in the literature. A lack of response in the biobank network to warrant standard optimized procedures for the isolation, characterization, and storage of EXOs will undoubtedly lead to a waste of resources and failure. This review is aimed at highlighting the increasing importance of EXOs for the clinic, especially in the cancer field, and at summarizing the initiatives taken to improve current isolation procedures, classification criteria, and storage conditions of EXOs as an effort to identify technological demands that biobank platforms face for the incorporation of EXOs and other extracellular vesicle fractions as valuable biospecimens for research.

## 1. Introduction

Both healthy and unhealthy cells secrete vesicles into the extracellular space. These extracellular vesicles (EVs) are majorly classified as exosomes (EXOs), microvesicles (MVs), retrovirus-like particles, and apoptotic bodies (APOs) according to their origin [1,2,3,4,5,6]. EVs entrap lipids, proteins and nucleic acids which can mediate varied functions while their membrane composition allows them to selectively deliver their content into particular surrounding and distal target tissues [7,8,9,10,11,12,13]. In particular, EXOs are currently defined as cup-shaped nanovesicles about 30–100 nm wide that originate within the endosomal network and can be found in most body fluids, including urine, saliva, blood, breast milk, and cerebrospinal fluid. They are of particular interest to the study of complex diseases such as cancer, neurodegenerative, cardiovascular, metabolic, and other complex diseases for their contribution to long-range intercellular communication and for their biomarker potential [7,8,9,10,11,12,13]. The observation that an increased production of EXOs is associated with disease, and the fact that EXO content also varies with disease state, gained researchers attention on the potential of EXOs as biomarkers and as vehicles to potentiate or spread disease [13,14]. In addition, as our understanding of the biology of EXOs intensifies, so does the range of principles to design EXOs and conjugated EXOs for their use as nanocarriers for immuno-therapeutics or vaccines, to carry angiogenesis modulators and for many other applications [15,16,17].

The term biobank has been defined in many ways [18]. Here, we adopt the following definition: a long-term storage and preservation facility that oversees the acquisition, processing, and distribution of high-quality biological specimens needed for future scientific investigation. Each sample deposited in a biobank has two main components: (1) the biological material collected, processed; and stored and (2) the database that contains information regarding demographic and clinical data for every sample in the inventory [19]. Peripheral blood, plasma, serum, isolated peripheral blood mononuclear cells (PBMCs), other blood-derived cell types, solid tissues, urine, saliva, RNA, and DNA are among the most common fractions being processed and stored in biomedical research biobanks. The increasing relevance of EVs, and in particular of EXOs in cancer, metabolic diseases, and other complex diseases made us wonder whether the incorporation of EXOs as a fraction to be consistently obtained and preserved in biobanks following consensus good manufacturing practice (GMP) guidelines could translate into a significant advance in the knowledge, diagnoses and treatment of these particular diseases.

Here we briefly review the controversies regarding EVs nomenclature and suggest actions for the discovery of functional markers that might aid in their classification. In addition, we present the major fronts of EXO-based research according to their potential application in the clinic, as biomarkers, delivery vehicles and/or therapeutic targets, which highlights EXOs’ increasing importance in medicine. To finish, we describe the technological uncertainties that limit progression of the field and propose the participation of the biobanks as a strategy to minimize these limitations.

## 2. EXOs Definition, Nomenclature, and Classification Criteria

EXOs’ nomenclature as a particular class of EVs, which also include apoptotic bodies (APOs) and microvesicles (MVs), is not consistent throughout the literature [2,20,21]. Despite efforts invested by the International Society of EVs (ISEV) to unify the nomenclature and the methodologies of EVs, no consensus criteria has been so far established [2]. In fact, the ISEV promotes the use of the term EVs instead of EXO due to the confusion created by different nomenclature systems based on varied criteria, *in vitro* studies and outdated isolation and detection techniques [20]. ISEV proposal is based on the following recent findings: (1) EVs indistinguishable from EXOs were shown to be released directly from the plasma membrane of cells; (2) diameters of EXOs were reported up to 250 nm; and (3) proteins such as tetraspanins were shown not to be unique for EXOs. In particular, tetraspanins CD9, CD63, and CD81 which were thought to be EXO-specific have been found in APOs [2,21] and CD9 and CD81 are among the most common markers in EVs [22]. ISEV EVs’ nomenclature is supported by the International Society of Thrombosis and Hemostasis (ISTH) and yet the most commonly used classification criteria for EXOs in the literature continues to be their size restricted to 30–100 nm in diameter, although considered to be variable and surpass this range limits; their cup-shaped morphology, which is known to be an artifact of TEM (transmission electron microscopy) fixation [23]; and the presence of the tetraspanin protein pan-markers CD9, CD63, and CD81, already known not to be EXO-specific [2,21]. Morphology assessment by TEM imaging is costly and time consuming and does not provide specific marker information. Alternative methods based on tunable pulse sensing strips (TRPS) and nanoparticle tracking analysis (NTA) do not provide evidence for the vesicular nature of the particles and require special instrumentation not available in all laboratories [24].

In an effort to identify functional EV specific markers proteomic databases of EVs such as Vesiclepedia [25], EVpedia [22], and ExoCarta [26] have been created. In general, it has been found that EVs are highly rich in cytoskeletal-, cytosolic-, heat shock-, and plasma membrane proteins, as well as in proteins involved in vesicle trafficking. Intracellular organelle proteins seem less abundant [2,22,25,26].

**Figure 1:**
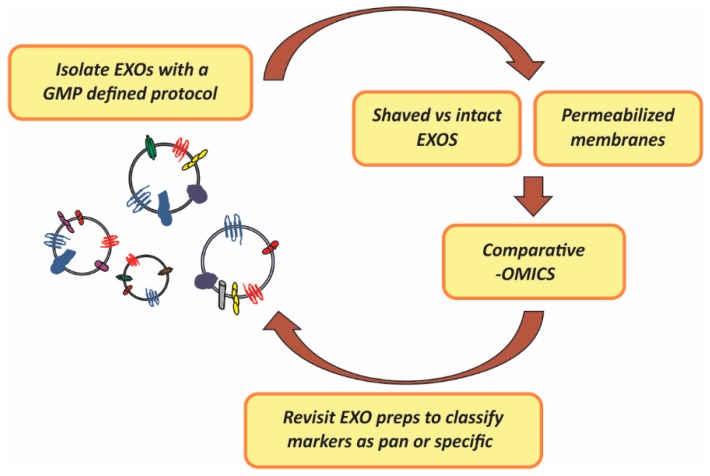
Strategy proposed for the identification of EXOs (exosomes) pan (general) and specific surface markers to serve as classification and functional subtyping criteria. GMP (Good Manufacturing Practices); preps (preparations); -OMICs (proteomic, lipidomic and transcriptomic analysis).

In summary, as it occurs in the case of lymphocyte subpopulations and other complex analytes, EXO preparations might present uniform particle size and morphology and still be a functional mixed entity. We propose rigorous profiling of EXOs membrane composition and landscapes prepared with a particular GMP isolation method which, importantly, should take into consideration potential technical problems derived from the presence of high abundant contaminants such as albumins and immunoglobulins commonly found in preparations of EXOs from cell culture supernatants, plasma or serum, aimed at establishing consensus classification subtyping criteria [27]. The strategy for this systematic in-depth analysis includes a retrospective visit to the starting fractions as a way to validate the identified membrane markers as ubiquitous (pan) or present in particular subfractions (specific). Figure 1, below, illustrates the strategy proposed which is mainly based on mass-spectometry proteomic analysis of either EXO membranes or differential profiling of intact *vs.* “shaved” EXOs (enzyme treated EXOs) and which should lead to functional marker discovery.

Preparation of EXOs from stored plasma or other protein-rich fluids subjected to freeze-thaw cycles might have hampered EXO surface marker identification. Thus, -OMIC studies of EXOs prepared from fresh fractions are encouraged.

## 3. EXOs as Biomarkers

Three mechanisms have been described to explain how EXOs operate as communicating vehicles: receptor mediated uptake, direct fusion with target cell plasma membranes, and endocytosis by phagocytosis. Processes that are all heavily regulated [8,9,10]. Contrary to signaling molecules in body fluids, the intra-vesicle cargo is protected from degradation by the EXO lipid bilayer. In addition, the fact that EXOs are enriched in specific proteins, lipids, and RNAs, while lacking others, indicates the existence of specialized mechanisms that control the sorting of signaling molecules [2,9,11], making EXOS attractive candidate biomarkers [12].

Current experimental evidence supports a central role of EXOs in cancer development, progression, metastasis, and drug resistance through: (1) promotion of carcinogenesis and tumor growth; (2) angiogenesis; (3) modulation of the tumor microenvironment; (4) modulation of immune responses; and (5) induction of mechanisms to acquire therapy resistance [7,28,29,30,31]. The precise mechanisms implicating EXOs to this plethora of effects are not completely understood. However, it is known that EXOs transport oncogenes and onco-miRNAs to other cells [28,31]. For example, in epithelial cancers, EXOs have been found to contain inducers of the epithelial-to-mesenchymal transition (EMT) such as TGFβ, TNF-α, IL-6, TSG101, AKT, ILK1, β-catenin, hepatoma-derived growth factor, casein kinase II, annexin A2, integrin 3, caveolin-1, and matrix metalloproteinases [32]. Cancer-derived EXO contents account for angiogenesis and pre-metastatic niche formation [29,33,34]. They also promote fibroblast growth at the tumor site, creating a desmoplastic reaction that limits the delivery of systemic therapy [35].

In addition to direct transfer of miRNAs [35] or miRNA-mediated immunosuppression [36,37], EXOs may promote carcinogenesis and drug resistance through alternative mechanisms. For example, once the cancer drug reaches the tumor cells, the drug could be redirected to EXOs, thus decreasing its intracellular availability; or EXOs could efflux receptor-antibody complexes during treatment, as in the case of Her-2 receptor-antibody complexes, thus inhibiting the anti-proliferative effects of the drug. Other yet possible mechanisms include: (1) turning tumor cells toward a rescue pathway when the primary tumorigenic pathway is locked; (2) epigenetic switches or inhibition of expression of tumor-suppressor proteins by miRNAs; (3) presence of cancer stem cells with high drug resistance; and (4) reduction in the penetration of anti-cancer drugs. Based on the above characteristics, EXOs seem to fit with the criteria for useful clinical biomarkers for the diagnosis and prognosis of cancer and to determine patient’s response to therapy [38,39,40].

The higher amount of EXOs in blood compared to circulating cancer stem cells should result in an increased sensitivity in EXO-based testing. In addition, the protective environment that EXOs provide to proteins and miRNAs suggests that EXO-based assays should have additional quantitative advantages over those based on circulating miRNAs or DNA. Moreover, EXOs’ miRNA content could reveal the nature of the primary tumor since miRNA profiles are tissue dependent [41], thus allowing establishment of new criteria for stratification of tumors. In particular, EXO-based diagnostic tests for cancer could be envisioned as a less invasive, specific, and sensitive “liquid biopsy” alternative providing real-time patient information [42].

However, for a molecule or particle to be classified as biomarker, the scientific community requires: (1) proof of concept; (2) experimental validation; (3) analytical performance validation (sensitivity, specificity, robustness, accuracy, *etc.*); and (4) protocol standardization. So far, EXOs have been studied in proof-of-concept and experimental validation studies [12,42,43,44], but we are not aware of commercially approved EXO-based cancer diagnostic tests for implementation in the clinic.

## 4. EXOs as Therapeutic Vehicles

EXOs have also been proposed as a natural tool for the treatment of cancer and other diseases. Given their ability to cross the blood brain barrier and selectively reach target organs and cells, they are proposed as attractive drug delivery vehicles [45,46,47]. EXOs provide certain innate advantages over artificial delivery systems: they are derived from natural sources, are immunologically inert, possess intrinsic ability to cross biological barriers and appear to be well tolerated even after application of repeated doses [48]. In order to consider EXOs for drug delivery, the following parameters need to be evaluated: (1) drug loading capacity, including protein and genetic material; (2) toleration by the human body with appropriate circulation times; (3) membrane penetration capability, intrinsic homing ability; and (4) amenability to membrane modifications. In addition the following issues need to be optimized: (1) cellular source (dendritic cell, bone marrow, mesenchymal stem cells (MSCs), induced pluripotent stem cells or others); (2) isolation and purification techniques (ultrafiltration, serial ultrafiltration, sucrose gradient, magnetic bead affinity capture); (3) loading techniques (electroporation, transfection of parent cell, exploitation of endogenous loading mechanisms) and (4) targeting/delivery strategies (antigen-mediated targeting, intravenous, intranasal, fusion of target peptide to EXO surface protein, or chemical modification of EXO surface) [16,49].

Mesenchymal Stem Cells (MSCs) are readily available from tissues like adipose tissue, liver, muscle, amniotic fluid, placenta, umbilical cord blood, and dental pulp, among others [50] and can be effectively expanded *ex vivo*. This characteristic has opened the possibility of using MSCs as a source of tumor-targeting EXOs [51]. Fitting with MSC characteristics, MSC-derived EXOs are expected to be non-immunogenic in allogeneic treatments and to possess intrinsic therapeutic properties in reducing tissue injury. Katsuda *et al.* studied the potential use of adipose-derived MSCs to deliver EXO-associated neprilysin to target tissues and concluded that MSC are cost-effective producers of EXOs for drug delivery [52,53]. In their work, they first identified adipose MSCs as a rich source of EXO-associated neprilysin compared to bone-marrow-derived EXOs. They then co-cultured adipose tissue MSCs with neuroblastoma N2a cells, which express human Aβ peptide at high levels. Their results showed that adipose MSC EXOs transferred neprilysin to N2a cells in a unidirectional fashion decreasing the levels of Aβ peptide. These exciting results illustrate the potential of MSC-derived EXOs to deliver their cargo specifically and functionally. For Alzheimer’s patients it represents a long-time expected effective treatment against the disease. Likewise, EXOs could be loaded with tumor-suppressive RNAs as a strategy for controlling tumor growth. Furthermore, EXO-based therapy has been proposed as immune therapy against tumors through their loading with cancer antigens that could lead to effective vaccination programs [20,46,54]. Manipulation of EXOs, without jeopardizing their biological properties, will constitute an additional challenge.

## 5. EXOs as Therapeutic Targets

To assess the impact of EXOs in clinical research, we searched clinical trials web page [55] using “exosome” as the search term. This search engine identified 22 registered government and private trials worldwide. One of them had been withdrawn, 16 are currently recruiting participants, two have not started yet and two of them are completed but without available results, indicating the novelty of this avenue. A high percentage (68%, corresponding to 15 out of the 22 studies) were cancer-related while two were related to diabetes, two to blood coagulation and tissue healing, one to Parkinson disease, one to cardiovascular disease, and one to Port-Wine Stain disease. Most studies have a diagnostic purpose, although a few focus on treatment with plant EXOs. It is also interesting that when we searched for “extracellular vesicles”, only one study was identified and it was not part of the “exosome” search. The number and wide variety of clinical trials in this area indicate the high impact of EXOs in clinical cancer research.

From the biosecurity point of view, we find it worrisome that clinical trials on EXOs have started even before a consensus definition on EXOs has been achieved. We identified several gaps in knowledge that need to be addressed, such as, definition of more specific and functional subgroups, and consensus isolation techniques with preservation of biological characteristics among other. We propose that filling these gaps will allow to provide better standardization of type and content of EXO fractions, leading to more controlled and directional uses of EXOs in the clinic. We envision that in a clinical setting, and due to the plasticity of EXOs landscape to environmental and pathological cues their specific markers could be used to obtain a patient Exogram (particular combination of EXO-associated markers for a particular individual at a determined moment) with the purpose of precisely diagnosing or monitoring the response to treatments as part of precision medicine programs. Since the associated patient clinical data might be key for identifying relevant Exogram markers, we consider that biobanks can provide the EXO-based research field an excellent opportunity to achieve its maximum development by providing the ideal legal infrastructure for a service platform connecting researchers and clinicians.

As an apparent controversy to the results showing that tumor-derived EXOs promote tumor progression, metastasis, and drug resistance [56,57], strategies to remove cancer EXOs systemically from cancer patients are not contemplated in any of the currently registered clinical trials. We recently reported that an in-depth evaluation not only of the tumor-derived EXOs contents, but also of the EXOs’ surface landscapes is needed in order to determine whether EXOs represent a threat or an advantage for a particular patient at a particular time during his/hers clinical management period [27].

## 6. Should EXOs Be Biobanked?

Given the need to address basic questions regarding EXOs isolation, preservation, and characterization, we present the impact that biobanking EXOs could have in this growing field of research.

### 6.1. EXO Standard Isolation Procedures

The most commonly used method of EXO preparation from clinical samples and cell culture supernatants is based on a series of successive centrifugations [58,59,60,61]. Interestingly, Lobb *et al.* showed that concentration of the sample using ultrafiltration devices based on centrifugation compared to pressure-driven devices is more appropriate and achieved in a reasonable amount of time. [60]. The requirement for techniques with high specificity, amenable to a relatively fast assay using a minimal amount of body fluid, which obviously cannot rely on costly time-consuming gradient purification techniques, led to the development of alternative procedures based on ultrafiltration and size-exclusion liquid chromatography, immunoaffinity, microfluidics, gradient methods, and polymeric precipitation [61,62,63,64,65]. Yields obtained with these alternative methods are variable, and in the case of immunoaffinity-based procedures, purity is achieved at a high cost. More recently, an efficient method based on polyethylene glycol/dextran aqueous two phase system (ATPS) has been used for the successful purification of EXOs [66]. Although all these methods represent a great advance for analytical profiling downstream applications, they present limitations for EXO functional assays. Further, the preparations obtained might contain a mixture of EXOs with varied properties diluting out the subgroup and, therefore, the markers of interest. Sub-fractionations of EV subgroups could be also achievable using compounds reactive with their surface, such as heparin [67] which, however, might interfere with downstream applications [68]; by charge separation or isoelectric focusing [69,70]; size (along with other chemical characteristics) or field flow fractionation techniques [2,71]. Not surprisingly, the method of choice for isolation of EVs impacts down-stream analysis of their cargoes [72,73].

To quantitate yields of EXOs, many studies use total protein content based on Micro BCA Protein Assay as an integral step to quantitate and normalize the amount of EXOs in preparations prior to performing downstream analysis. An important limitation of using total protein content in addition to the reported interference of glucose and tryptophan [24] is that soluble proteins and protein complexes are prevalent in body fluids and culture media. Furthermore, protein aggregates co-purify with different EXOs [24,74] and vesicle membranes may rupture causing a loss in protein cargo and therefore lead to study biases putting their significance into question.

Thus, studies aimed at comparing yields, purity, and integrity of EXOs according to the isolation procedure are at need. In this sense, Kalra *et al*. thoroughly compared alternative techniques for EXO isolation from human plasma and showed that the density gradient method was superior to ultracentrifugation [75]. More studies of this type for EXOs from other sources are granted.

### 6.2. EXOs Functional Marker Validation

As mentioned in Section 2, several features for the characterization of EVs have been proposed. Some are based on EVs physical characteristics and others on their composition. EVs cannot be visualized in most microscopic platforms, thus, electron microscopy approaches including whole mount electron microscopy, scanning electron microscopy, TEM, and electron tomography are required [2,73,74,76]. These approaches are laborious and technically challenging. Alternative methods include techniques based on laser acquisition data such as particle tracking analysis, including NanoSight and Particle Metrix [77] but this morphologic analysis does not provide functional information of the isolated EXO-fraction. In addition to the CD63, CD9, and CD81 unspecific tetraspanin markers, other markers of EXO interior content (Acetyl-CoA acetylcholinesterase, Annexins, TSG 101) have been suggested as surrogate functional markers [2,72,73]. However, at present, no consensus pan-marker or functional specific markers have been convincingly established.

Functionality of the recovered EXOs is determinant for evaluating EXO potential applications in the clinic or in the biotechnology sector and therefore should be part of an EXO isolate characterization. Typically functions are evaluated by measuring EXO binding, delivery and/or release of their cargo to target cells. The strategies consist in fluorescently labeling EXOs by internal fusion proteins or by including tracking dyes for their visualization that is achieved by flow cytometry, live tracking, or other types of fluorescence-based imaging procedures. In particular, the PKH67 uptake dye assay has been used to show the capacity of EXOs isolated from the plasma of patients and stored at −20 °C for over 30 days, to fuse with LIM 1215 colorectal cancer cells [75]. Other researchers had shown benefits between different isolation methods, however, there has been no systematic approach to standardize functional protocols according to source and/or downstream applications [20,61,73,74]. To normalize function and in addition to total protein measurements, ELISA-based quantitation and Acetyl-CoA cholinesterase colorimetric assays are used [78,79]. However, these tests measure overall activities which include contribution from different EV types. Thus, the results on function are rough estimates.

More recently, Osteikoetxea *et al.* presented a simple and readily available lipid assay to complement the widely used protein assays, using protein to lipid ratio, lipid bilayer order, and lipid composition as parameters to differentiate extracellular vesicle subpopulations [24]. They showed that EXOs are characterized by highest membrane lipid order, while APOs and MVs showed low to intermediate, partially overlapping lipid order reflecting important differences that can impact vesicle subpopulations signaling pathways, fitting with the fact that high membrane lipid order is typically found at the immunological synapse, sites of cell adhesion, viral entry, and budding. This total lipid assay is simple and fast requiring only 30 min of incubation, and 0.5 μg protein containing EVs either in dry pellet or in up to 50 μL volume; and at concentrations >50 μg/mL lipid show low variability and good accuracy [24]. The authors also found a smaller increase in the protein to lipid ratios than what was expected based on geometry (surface area to volume ratio) in EXOs which may either represent different cargo packing densities or different molecular composition of the different subtypes. EXOs are also known to be particularly enriched in cholesterol and GM1 gangliosides [24,80,81]. However, this approach had been challenged by the fact that this ratio can changed in a variety of physiological, as well as disease states, limiting the application of this concept.

### 6.3. EXOs Optimized Storage Conditions

There are no strictly defined conditions for storing/isolating EXOs. The only regulated guidelines found are those established by ISTH for the isolation of platelets-derived EVs [82]. There is no specific information regarding the effect of anticoagulants in the collection and storage of EXOs, neither optimal time, temperature, storage period, freezing-thaw cycles, thawing conditions, or other storage variables have been thoroughly evaluated.

The study by Kalra *et al.* we have previously mentioned in this review [75], included the evaluation of the stability of EXOs at different temperatures finding that although at 90 days all samples were stable, there was an advantage in storing EXOs at −80 °C. However, this may vary for EXOs prepared from different sources and/or particular isolation procedures. In addition, the particular lipid composition of EXOs [2,24,80,81], which is source dependent as well, is expected to have an impact on optimal cryopreservation procedures, raising an additional challenge for EXO biobanking.

## 7. Role of Biobanks in EXO-Based Basic and Clinical Research

The science of biobanking focuses on the standardization of procedures by which samples are collected, processed, stored, and distributed. In addition, rigorous annotation of information regarding the “life cycle” of the specimen is crucial for the knowledge of its integrity and to achieve consistency throughout research studies. Annotation starts with the collection of information related to the donor and continues with detailed additional information on the acquisition, transport, processing, and storage of the sample. Pre-analytical variables allow for standardization of conditions that result in sample quality homogeneity and permit correct sample classification [83]. Analysis of the literature on EXOs showed a lack of information regarding these variables. This, in addition to the inconsistency to define EXOs and the failure to establish and implement standard GMP protocols for the isolation and storage of EXOs, makes us think there is an urgent need for biobanks to assume an active role in this research area.

**Figure 2:**
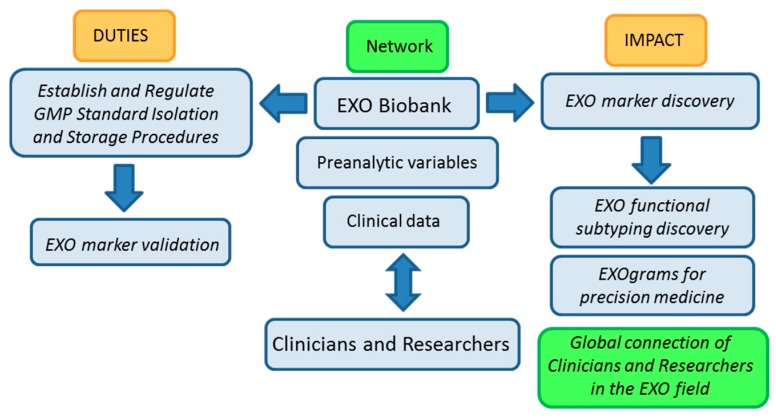
Biobanking of EXOs (exosomes) workflow: duties and impact on the advance of EXO-based research. GMP (Good Manufacturing Practices).

Biobanks already store biological body fluids for different purposes. If designed to prepare and store EXOs at a large scales, by their intrinsic nature, they will be able to support studies aimed at comparing isolation, detection, and storage protocols, allowing for the establishment of optimal standardized GMP guidelines based on well-annotated quality samples. The availability of high-quality EXO samples should help in turn, further understand EXOs’ true biology. In addition, EXO-based clinical personalized applications which ultimately rely on: (1) discrimination and precise diagnosis; (2) targeted therapies of choice for each patient; (3) dose adjustment methods to optimize the benefit-risk ratio of treatment; (4) biomarkers of efficacy, toxicity, treatment discontinuation, relapse, *etc.* will benefit from EXO biobanking activities if coordinated with those of pathologists and clinicians at hospital and health care centers. Figure 2, below, shows a work-flow diagram that highlights the expected impact of integrating biobank operation and networking in the EXO field as it transitions from the bench to the clinic.

The basic nature of biobanking research activities related to standardization of protocols and evaluation of samples in terms of isolation yields, pre-clinical parameters, factors that affect characterization and storage of samples among other, argues in favor of our proposal for biobanks playing a central role in the advance of EXO-based research and in the development of EXO-based clinical applications.

## 8. Conclusions

There is an urgent need for the establishment of a consensus classification criteria and nomenclature in the EVs field. The techniques for detection, isolation, and storage of circulating EXOs are still immature needing optimization and standardization. The use of unified markers that thoroughly characterize isolated vesicle fractions as functional EXO subsets results is imperative. A strategy to identify such surface EXO biomarkers for the development of Exograms as a clinical advance in precision medicine is presented.

Biobanks, as institutions applying standardized GMPs for the sampling, processing, and storage of human biological samples are proposed as the ideal legal infrastructure acting as a service platform to overcome present EXO-research hurdles and to expedite the advance of EXO-based diagnosis and treatments for the upcoming era of precision medicine.

## Abbreviations

EVs: Extracellular Vesicles

EXO: Exosome

MV: Microvesicles

APOs: Apoptotic Bodies

PBMCs: Peripheral Blood Mononuclear Cells

MSCs: Mesenchymal Stem Cells
